# Awareness Among Primary Care Physicians Regarding the Alarm Symptoms and Signs of Rhinosinusitis

**DOI:** 10.7759/cureus.46114

**Published:** 2023-09-28

**Authors:** Yahya A Fageeh, Muteb S Alsuwat, Yazeed A Almansouri, Abdulrahman H Alsuwatt, Faisal T Almalki, Abdullah A Al Shehri

**Affiliations:** 1 Otolaryngology - Head and Neck Surgery, College of Medicine, Taif University, Taif, SAU; 2 Medical School, College of Medicine, Taif University, Taif, SAU; 3 General Practice, College of Medicine, Taif University, Taif, SAU

**Keywords:** general practitioners, primary healthcare, intracranial complications, orbital complications, fungal rhinosinusitis, chronic rhinosinusitis, acute rhinosinusitis

## Abstract

Background

Rhinosinusitis is a common condition. Primary care physicians (PCPs) play a vital role in diagnosing and managing rhinosinusitis, including identifying alarm symptoms and signs. However, limited research exists on PCPs' awareness of these alarm symptoms.

Objectives

This study aimed to assess the awareness of PCPs in Saudi Arabia regarding the alarm symptoms and signs of rhinosinusitis and identify knowledge gaps.

Methodology

A descriptive cross-sectional study was conducted among 153 PCPs in Taif, Saudi Arabia. An online questionnaire covering demographic data and multiple-choice questions on alarm symptoms and signs of rhinosinusitis was administered. Knowledge level was assessed based on the score of the responses to knowledge items. Data analysis was performed using IBM SPSS software (IBM Corp., Armonk, NY).

Results

The study revealed a low awareness of alarm symptoms and signs of rhinosinusitis among PCPs. Knowledge gaps were observed in recognizing symptoms and appropriate referral pathways. Participants showed inadequate awareness of severe headaches, frontal swelling, anosmia, cacosmia, and nasal bleeding or crustation as alarm symptoms. The average knowledge score was 4.57 ± 1.91 out of 10.

Conclusion

The study highlights the need to improve PCP awareness of alarm symptoms and signs of rhinosinusitis in Saudi Arabia. Educational programs should be developed to address knowledge gaps and enhance understanding of guidelines, facilitating early identification and referral of severe cases and improving patient outcomes.

## Introduction

Rhinosinusitis is a medical condition that involves the inflammation of the mucosa in the nose and paranasal sinuses. Typical symptoms include nasal congestion, obstruction, discharge, facial pain, and loss of smell. There are two main types of rhinosinusitis: acute and chronic. Acute rhinosinusitis (ARS) typically lasts less than 12 weeks, while chronic rhinosinusitis (CRS) persists for 12 weeks or more [1,2]. 

The common cause of ARS is a virus, but there are cases where it is caused by bacteria, which results in more severe symptoms. This type of ARS is known as acute bacterial rhinosinusitis (ABRS) [3-5]. Chronic rhinosinusitis can have a variety of causes, including allergies, structural abnormalities, and immune system dysfunction [1].

Rhinosinusitis is a common condition that affects millions of people worldwide. It comprises many primary healthcare visits and substantial healthcare expenses [6].

The diagnosis of rhinosinusitis is typically based on the patient's symptoms and a rhinoscopy [1]. Treatment for ARS typically involves over-the-counter medications, such as decongestants, nasal saline sprays, and pain relievers [4]. However, broad-spectrum antibiotics are cardinal in treating ABRS to prevent complications, especially in children [7,8]. Treatment for CRS is quite complex and involves using medications such as intranasal and systemic steroids, antibiotics, saline irrigation, and antihistamines. Depending on the response to medical treatment and the findings of the examination and CT scan, endoscopic sinus surgery (ESS) may also be necessary [1,2]. 

Rhinosinusitis can give rise to various complications affecting the orbits, intracranial region, or bones. These complications can manifest as periorbital swelling, redness, eye displacement, vision problems, severe headache, frontal swelling, neck stiffness, altered mental state, and neurological deficits [9,10]. 

Fungal rhinosinusitis is a specific type of rhinosinusitis caused by a fungal infection, which can be invasive or non-invasive, depending on the extent of tissue penetration and the immunity of the affected individual. Fungal rhinosinusitis can progress to involve the orbit or brain, leading to severe complications [11].

The diagnosis and treatment of rhinosinusitis and its complications can be challenging. It becomes crucial to identify severe cases early on and ensure appropriate management by specialists [10]. Therefore, it is paramount for primary care physicians (PCPs) to possess the ability to accurately diagnose rhinosinusitis and determine when referral to a specialist is necessary [12]. However, the effectiveness of early intervention heavily relies on the awareness and knowledge of PCPs regarding the symptoms and signs associated with rhinosinusitis, particularly those indicative of severe and complicated cases [13]. Unfortunately, we could not locate studies examining PCPs' awareness in Saudi Arabia. Additionally, the international studies we came across have only assessed the understanding of PCPs in referring patients with severe and complicated rhinosinusitis without exploring their awareness of early alarming symptoms necessitating immediate referral [13-15].

Therefore, this study's primary aim was to evaluate general practitioners' awareness regarding the alarm symptoms and signs associated with rhinosinusitis. The study will provide valuable information about the level of awareness of PCPs in Saudi Arabia regarding alarm symptoms and signs of rhinosinusitis. This information will be used to develop educational programs to enhance the diagnosis and management of rhinosinusitis, ultimately leading to better patient outcomes.

## Materials and methods

Study design 

We conducted a descriptive cross-sectional study from January 2022 to January 2023 at primary healthcare centers in Taif, Saudi Arabia. The study included 153 general practitioners who work as PCPs. We excluded specialists and those certified in otorhinolaryngology, neurology, or ophthalmology. 

An online questionnaire was sent to participants' phone numbers, and they were invited to take part in the survey. The study's purpose was explained to them before they participated. The questionnaire included both open-ended and closed-ended questions, which were formulated based on previous studies.

The questionnaire

The questionnaire was developed from a study conducted in the Netherlands in 2011 and created by the authors of the European Position Paper on Rhinosinusitis and Nasal Polyps (EPOS) [14]. Modifications were made to accommodate the research outcomes according to EPOS 2020 guidelines [1]. The questionnaire was in English and consisted of two parts. The first part gathered information on demographic data and characteristics of participants, including age, gender, and practice duration. The second part consists of multiple-choice questions for different categories of rhinosinusitis, including complications and alarm symptoms and signs. They were asked when to refer these patients to the specialist for each condition. The participant's knowledge level was assessed based on their score on the knowledge items. If they scored over 75%, they were considered to have good knowledge, while a score between 50% and 75% indicated fair knowledge. A score below 50% was classified as poor knowledge.

Data analysis

Data analysis was carried out using the IBM SPSS statistical software version 26 (IBM Corp., Armonk, NY). The relationship between variables was examined using the Chi-squared test (χ2). Qualitative data were presented in numbers and percentages, while quantitative data were expressed as mean and standard deviation (mean ± SD). A p-value of less than 0.05 was considered statistically significant.

Ethical considerations 

The study received ethical approval from the Research Ethical Committee of Taif University, Saudi Arabia, with reference number 43-140. In order to ensure privacy, the answers given in the questionnaire were kept anonymous.

## Results

The study included a total of 153 PCPs from various primary care clinics. Most participants were male (58.8%) and had less than five years of clinical experience (Table 1).

**Table 1:** Distribution of the PCPs included in the study according to their age, gender, and practice duration (n = 153)

Variable	No. (%)
Age (years)	
<30	86 (56.2)
31-34	52 (34)
35-39	10 (6.5)
40-44	1 (0.7)
≥45	4 (2.6)
Gender	
Female	63 (41.2)
Male	90 (58.8)
Practice duration (years)	
< 5	97 (63.4)
5-10	45 (29.4)
10-15	6 (3.9)
15-20	2 (1.3)
20-25	3 (2)

This study investigated the knowledge of PCPs on the appropriate referral criteria for patients with rhinosinusitis (Table 2). 

**Table 2:** Distribution of the PCPs included in the study according to their response to knowledge items about alarm symptoms and signs of rhinosinusitis (n = 153) *: the correct answer

Variable	No. (%)
a. Patients with moderate rhinosinusitis symptoms for< 12 weeks	
After 48 hours, with no effect of intranasal corticosteroids or antibiotics	2 (1.3)
After a ten-day course of antibiotic treatment, which did not work	11 (7.2)
Always refer them to a specialist right after diagnosis	27 (17.6)
Never refer them to a specialist	15 (9.8)
When no improvement occurs after 14 days of treatment	69 (45.1)
When no improvement occurs after six weeks of treatment*	29 (19)
b. Patients with moderate rhinosinusitis symptoms for≥12 weeks	
After 48 hours, with no effect of intranasal corticosteroids or antibiotics	8 (5.2)
After a ten-day course of antibiotic treatment, which did not work	18 (11.8)
Always refer them to a specialist right after diagnosis	46 (30.1)
Never refer them to a specialist	1 (0.7)
When no improvement occurs after 14 days of treatment	43 (28.1)
When no improvement occurs after six weeks of treatment*	37 (24.2)
c. Patients with a fever >38 °C, severe facial pain, and purulent nasal discharge	
After 48 hours, with no effect of intranasal corticosteroids or antibiotics	25 (16.3)
After a ten-day course of antibiotic treatment, which did not work*	42 (27.5)
Always refer them to a specialist right after diagnosis	33 (21.6)
Never refer them to a specialist	6 (3.9)
When no improvement occurs after 14 days of treatment	33 (21.6)
When no improvement occurs after six weeks of treatment	14 (9.2)
d. Patients with signs of sepsis	
After 48 hours, with no effect of intranasal corticosteroids or antibiotics	16 (10.5)
After a ten-day course of antibiotic treatment, which did not work	4 (2.6)
Always refer them to a specialist right after diagnosis*	117 (77.2)
Never refer them to a specialist	2 (1.3)
When no improvement occurs after 14 days of treatment	8 (5.2)
When no improvement occurs after six weeks of treatment	5 (3.3)
e. Patients with neurological signs	
After 48 hours, with no effect of intranasal corticosteroids or antibiotics	4 (2.6)
After a ten-day course of antibiotic treatment, which did not work	5 (3.3)
Always refer them to a specialist right after diagnosis*	131 (85.6)
Never refer them to a specialist	0 (0)
When no improvement occurs after 14 days of treatment	7 (4.6)
When no improvement occurs after six weeks of treatment	6 (3.9)
f. Patients with anosmia	
After 48 hours, with no effect of intranasal corticosteroids or antibiotics	10 (6.5)
After a ten-day course of antibiotic treatment, which did not work	4 (2.6)
Always refer them to a specialist right after diagnosis	44 (28.8)
Never refer them to a specialist	15 (9.8)
When no improvement occurs after 14 days of treatment	46 (30.1)
When no improvement occurs after six weeks of treatment*	34 (22.2)
g. Patients with cacosmia	
After 48 hours, with no effect of intranasal corticosteroids or antibiotics	12 (7.8)
After a ten-day course of antibiotic treatment, which did not work	11 (7.2)
Always refer them to a specialist right after diagnosis*	52 (34)
Never refer them to a specialist	1 (0.7)
When no improvement occurs after 14 days of treatment	46 (30.1)
When no improvement occurs after six weeks of treatment	31 (20.3)
h. Patients with orbital symptoms or signs	
After 48 hours, with no effect of intranasal corticosteroids or antibiotics	10(6.5)
After a ten-day course of antibiotic treatment, which did not work	9 (5.9)
Always refer them to a specialist right after diagnosis*	107 (69.3)
Never refer them to a specialist	1 (0.7)
When no improvement occurs after 14 days of treatment	14 (9.2)
When no improvement occurs after six weeks of treatment	13 (8.5)
i. Patients with severe headache	
After 48 hours, with no effect of intranasal corticosteroids or antibiotics	23 (15)
After a ten-day course of antibiotic treatment, which did not work	20 (13.1)
Always refer them to a specialist right after diagnosis*	30 (19.7)
Never refer them to a specialist	5 (3.3)
When no improvement occurs after 14 days of treatment	48 (31.3)
When no improvement occurs after six weeks of treatment	27 (17.6)
j. Patients with nasal bleeding or crustation	
After 48 hours, with no effect of intranasal corticosteroids or antibiotics	17 (11.1)
After a ten-day course of antibiotic treatment, which did not work	14 (9.1)
Always refer them to a specialist right after diagnosis*	48 (31.4)
Never refer them to a specialist	9 (5.9)
When no improvement occurs after 14 days of treatment	39 (25.5)
When no improvement occurs after six weeks of treatment	26 (17)
k. Patients with frontal swelling	
After 48 hours, with no effect of intranasal corticosteroids or antibiotics	14 (9.2)
After a ten-day course of antibiotic treatment, which did not work	16 (10.5)
Always refer them to a specialist right after diagnosis*	77 (50.3)
Never refer them to a specialist	2 (1.4)
When no improvement occurs after 14 days of treatment	26 (17)
When no improvement occurs after six weeks of treatment	18 (11.8)

The findings revealed that about a quarter of PCPs would refer patients with moderate CRS symptoms to a specialist after appropriate therapy. Similarly, for ARS, only 19% of PCPs would refer patients to a specialist if there was no improvement after appropriate therapy.

In cases of acute bacterial ABRS, only 27.5% of PCPs would refer patients if they did not improve after ten days on antibiotics. However, most PCPs would refer their patients immediately if they suspected sepsis.

Regarding rhinosinusitis and neurological symptoms, 85.6% of PCPs would promptly refer their patients to a specialist. However, for severe headaches, most of them tended to delay the referral.

When dealing with rhinosinusitis accompanied by orbital symptoms or signs, 69.3% of PCPs would promptly refer patients to a specialist. However, in the case of frontal swelling, only about half of PCPs would make an immediate referral.

Many participants did not adequately recognize anosmia as a symptom of rhinosinusitis. Furthermore, 35.3% consider it a reason for urgent or immediate referral. In contrast, about 21% of PCPs did not consider cacosmia an alarming symptom.

Furthermore, more than half of PCPs delayed referring patients with nasal bleeding or crustation to a specialist, and around 5.9% stated they would never refer them.

**Figure 1:**
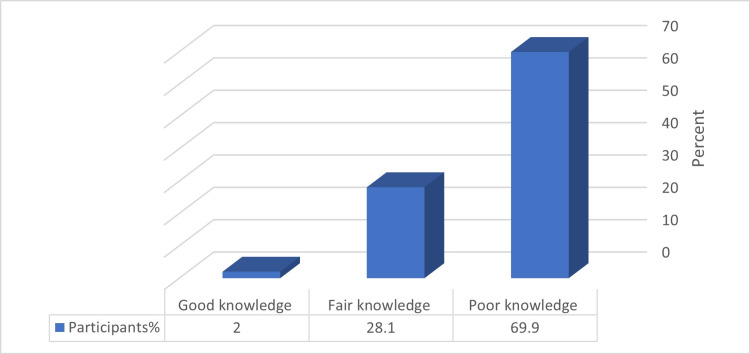
Percentage distribution of the participants according to their knowledge level about alarm symptoms and signs of rhinosinusitis (n = 153)

Most participants showed inadequate knowledge concerning the alarm signs and symptoms of rhinosinusitis (Figure 1).

The average score for participants' knowledge was 4.57 ± 1.91.

Finally, the study found no significant correlation between PCPs' knowledge level about alarm symptoms and signs of rhinosinusitis and their demographic characteristics or practice duration (Table 3).

**Table 3:** Relationship between the PCPs' knowledge level regarding alarm symptoms and signs of rhinosinusitis and their demographic characters and practice duration (n = 153)

Variable	Knowledge level			χ2	p-value
	Poor No. (%)	Fair No. (%)	Good No. (%)		
Age (years)					
<30	62 (57.9)	22 (51.2)	2 (66.1)	2.12	0.977
31-34	35 (32.7)	16 (37.2)	1 (33.3)		
35-39	7 (6.5)	3 (7)	0 (0.0)		
40-44	1 (0.9)	0 (0.0)	0 (0.0)		
≥45	2 (1.9)	2 (4.7)	0 (0.0)		
Gender					
Female	41 (38.3)	20 (46.5)	2 (66.7)	1.67	0.434
Male	66 (61.7)	23 (53.5)	1 (33.3)		
Practice duration (years)					
< 5	66 (61.7)	29 (67.4)	2 (66.7)	5.88	0.66
5-10	6 (5.6)	0 (0.0)	0 (0.0)		
10-15	2 (1.9)	0 (0.0)	0 (0.0)		
15-20	1 (0.9)	2 (4.7)	0 (0.0)		
20-25	32 (29.9)	12 (27.9)	1 (33.3)		

## Discussion

The results of this study reveal a low level of knowledge among PCPs in Taif, Saudi Arabia, concerning the alarm symptoms and signs of rhinosinusitis. The results highlight a significant gap in the understanding and application of the EPOS 2020 guidelines among the studied sample.

The majority of the participants in the study were young and had limited professional experience, which could explain some of the observed knowledge gaps. These findings align with similar studies conducted internationally [13,14]. 

The gaps in knowledge were particularly notable in comprehending the different types of rhinosinusitis, the criteria used for diagnosis, and the proper referral pathway.

A patient suffering from severe and complicated rhinosinusitis must be immediately referred to an otolaryngologist. It requires PCPs to possess a strong understanding of the condition, adequate communication skills, and a quick referral system [16,17].

Most participants acknowledge the significance of referring patients to specialists without delay when they experience neurological or orbital complications and sepsis. In such critical scenarios, timely referrals can significantly impact the patient's recovery [8,16]. However, there is a dearth of knowledge about severe headaches being a potential symptom of complicated rhinosinusitis, which requires immediate attention from a specialist [18, 19]. Additionally, only half of the participants knew that the presence of frontal swelling necessitated an urgent referral [20].

Rhinosinusitis can result in anosmia. However, some PCPs are unaware that anosmia is a rhinosinusitis symptom [1,2]. On the other hand, nearly half of the participants believe anosmia is severe and requires an urgent referral and immediate medical attention. This could lead to unnecessary referrals and cause undue anxiety for patients. Furthermore, such actions may overload the healthcare system.

Cacosmia could be a symptom of severe rhinosinusitis or fungal rhinosinusitis, which is considered an alarming symptom [1,11]. However, this study shows that about half of PCPs need to be made aware of the significance of cacosmia. This lack of knowledge may lead to delayed treatment for a severe condition.

The presence of bleeding or crusting may indicate an underlying condition such as granuloma, vasculitis, or malignant or nonmalignant growth. These require further evaluation by a specialist [21]. Unfortunately, most participants are not aware of this fact.

These findings suggest that while there is a considerable understanding of the need for immediate referral in severe cases, there is a lack of knowledge about the alarm symptoms and signs of these cases. This gap could lead to a delay in appropriate referrals and potentially worsen patient outcomes.

A significant limitation of this research was the lack of previous studies on alarm symptoms and signs of rhinosinusitis, which may have hindered the scope of the discussion. Another limitation was using a self-reporting questionnaire, which could have resulted in a recall bias.

## Conclusions

While the study results showed a reasonable level of knowledge in some areas, there is a clear need for further education and training to improve the awareness of PCPs regarding the alarm symptoms and signs of severe and complicated rhinosinusitis. Given the prevalence of rhinosinusitis and the critical role that PCPs play in its initial diagnosis and management, this is an essential area for further research and intervention. Future studies could evaluate the effectiveness of different educational interventions in improving the knowledge and clinical practice of PCPs in this area.
